# Brain-to-brain hyperclassification reveals action-specific motor mapping of observed actions in humans

**DOI:** 10.1371/journal.pone.0189508

**Published:** 2017-12-11

**Authors:** Dmitry Smirnov, Fanny Lachat, Tomi Peltola, Juha M. Lahnakoski, Olli-Pekka Koistinen, Enrico Glerean, Aki Vehtari, Riitta Hari, Mikko Sams, Lauri Nummenmaa

**Affiliations:** 1 Department of Neuroscience and Biomedical Engineering, School of Science, Aalto University, Espoo, Finland; 2 AMI Centre, Aalto NeuroImaging, School of Science, Aalto University, Espoo, Finland; 3 Helsinki Institute for Information Technology HIIT, Department of Computer Science, Aalto University, Espoo, Finland; 4 Department of Art, School of Arts, Design and Architecture, Aalto University, Espoo, Finland; 5 Turku PET Centre and Department of Psychology, University of Turku, Turku, Finland; University of California Los Angeles, UNITED STATES

## Abstract

Seeing an action may activate the corresponding action motor code in the observer. It remains unresolved whether seeing and performing an action activates similar action-specific motor codes in the observer and the actor. We used novel hyperclassification approach to reveal shared brain activation signatures of action execution and observation in interacting human subjects. In the first experiment, two "actors" performed four types of hand actions while their haemodynamic brain activations were measured with 3-T functional magnetic resonance imaging (fMRI). The actions were videotaped and shown to 15 "observers" during a second fMRI experiment. Eleven observers saw the videos of one actor, and the remaining four observers saw the videos of the other actor. In a control fMRI experiment, one of the actors performed actions with closed eyes, and five new observers viewed these actions. Bayesian canonical correlation analysis was applied to functionally realign observers' and actors' fMRI data. Hyperclassification of the seen actions was performed with Bayesian logistic regression trained on actors' data and tested with observers' data. Without the functional realignment, between-subjects accuracy was at chance level. With the realignment, the accuracy increased on average by 15 percentage points, exceeding both the chance level and the accuracy without functional realignment. The highest accuracies were observed in occipital, parietal and premotor cortices. Hyperclassification exceeded chance level also when the actor did not see her own actions. We conclude that the functional brain activation signatures underlying action execution and observation are partly shared, yet these activation signatures may be anatomically misaligned across individuals.

## Introduction

To successfully interpret each other’s actions and intentions, humans need to have similar-enough understanding of the external world. One prominent model based on monkey and human data proposes that the observer, while viewing others’ actions, automatically simulates or “mirrors” some aspects of motor activity of the actor, as is evidenced by activation of a frontoparietal brain network, including the premotor and primary motor cortices [1–4] during both performing and viewing an action. This shared sensorimotor information may subsequently enable the observer to mimic motor actions and sensations of another individual, supporting understanding of the other person’s actions or action goals [3, 5]. If the mirroring hypothesis of action understanding is true, then different actions associated with different motor codes in the actor’s brain should result in correspondingly different brain activation signatures in the observer.

Prior functional brain imaging studies using pattern-classification approach suggest that both action observation and execution are associated with action-specific neural fingerprints in the parietal, premotor, and lateral occipital cortices [6–12]. Moreover, shared brain activation signatures have been observed between executed and perceived actions in single individuals [13, 14]. Similar mechanisms were proposed for affective processing, as corresponding neural patterns were found during emotion observation and one's own emotional experience [15]. Also in line with the direct-matching hypothesis were the findings that somatosensory activation allowed successful classification of the type of observed touch [16]. Shared brain activity between two interacting individuals was also investigated in gestural communication [17, 18], showing similarities in temporal structure of brain activity involved in guessing the meaning of a gesture and gesturer’s brain activity in regions involved in mentalizing and mirroring. However, even though intraparietal activation patterns allowed successful classification of various observed or executed manual actions, these patterns were different for action execution and observation [6]. Overall, while there is evidence for shared brain activation signatures for action execution and observation in single individuals, it remains a question whether those activation signatures are shared across individuals, where one is performing, and the other is observing the action.

The overlap of neural activity patterns does not directly prove sharing of neural brain activation signatures for action observation and execution in the brains of two interacting individuals. Such sharing would be in line with a direct-matching mechanism, which proposes automatic generation of internal representations of the observed motor acts, thus allowing the observed actions to be directly mapped onto the observer’s motor system [3]. However, because individuals differ in functional and structural organization of their cerebral cortex, it is reasonable to assume that anatomically corresponding areas in the frontoparietal circuitry could differ in how they represent action execution in one and its observation in another brain. Recent work has shown that individual differences in functional and anatomical organization of the ventral visual cortex can be accommodated with a high-dimensional common-space “hyperalignment” model [19, 20] that improves the group-level estimates of haemodynamic responses. Accordingly, executing and observing a motor action could result in information-wise similar patterns of neural activity in the corresponding brain regions of the actor and the observer, yet these patterns may fail to match in the common coordinate space. Such idiosyncratic brain activation signatures in actors and observers can however be mapped to shared space using functional realignment techniques.

Here we hypothesized that the brain activation patterns of an action observer can be reliably predicted from the brain activity of the individual performing the actions after the observer’s and actor’s brains are functionally aligned. We developed a novel hyperclassification approach, which combines functional realignment, based on a common functional space between performing and observing action, with between-subjects classification to reveal the shared action-specific neural codes of action execution and observation across two different brains. The ‘actor’ subjects performed four different hand actions, while their haemodynamic brain responses were measured with functional magnetic resonance imaging (fMRI). The actions were videotaped and shown subsequently to 'observer' subjects during fMRI. The pattern classifier was trained on the actor’s data and tested with the observer's data realigned to the actor’s space. We specifically tested whether the functional realignment would allow accurate classification of the observed actions on the basis of motor signatures of the corresponding actions.

## Materials and methods

### Participants

Twenty-two healthy right-handed adults with normal or corrected to normal vision and normal hearing (self-reported) volunteered for the study. The subjects were divided into ‘actor’ and ‘observer’ subgroups. The actors included two female individuals (ages 23 and 29 years), and the observers included twenty individuals (10 females and 10 males; mean age 28 years, range 22–56 years). Subjects had no history of neurological or psychiatric diseases or current medication affecting the central nervous system. All subjects were compensated for their time, and they signed informed consent forms. The research plan and the informed consent forms were approved by the Aalto University Research Ethics Committee.

### Experimental setup for actor subjects

**Fig 1:**
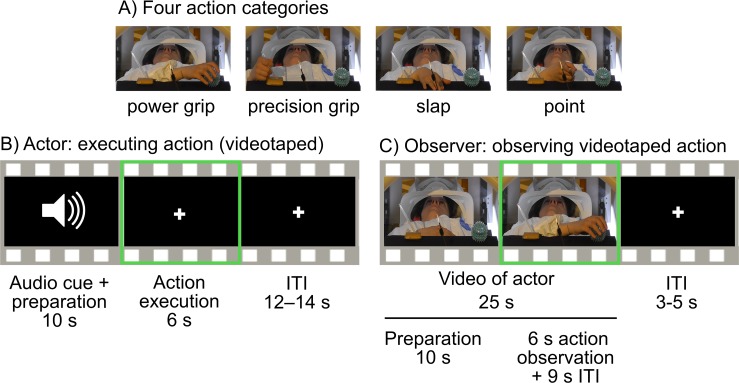
Experimental design and sample trials of action execution and observation. A) The actor performed four different actions in the scanner. An actor in the control experiment kept her eyes closed and could not see her own hand actions. B) Trial structure for the actor subjects. C) Trial structure for the observer subjects. Green outline shows the trial portions used for classification. Photographer identified themselves and the purpose of the photograph to the person shown in the photograph, and they agreed to have their photograph taken and potentially published.

Two female ‘actor’ subjects performed four different hand actions (Fig 1A) with their right hand while being scanned with fMRI. The actions included two object-directed actions (*power grip* of a soft spiky ball and *precision grip* of a plastic pen) and two non-object directed actions (soft *slap* on the table; and a *pointing* gesture). In the power grip, a whole-hand grasping movement was used to grab a soft ball with the fingers flexed to form a clamp against the palm. In the precision grip, the actors used opposition of thumb and middle and index finger fingers to grab a vertically standing pen. In slapping, an open palm was put softly on the table. Pointing constituted of pointing towards the front of the scanner bore with the index finger. All actions were performed over a black wooden table placed above the actor’s hip, but not touching the body, so that no tactile contamination could rise from table movements. The actors practiced the actions before the experiment started. A mirror box attached to the head coil allowed the actors to see the table. A green LED light was positioned in the middle of the actor’s field of view to cue trial onsets and offsets. Auditory cues were delivered with Sensimetrics S14 insert earphones (Sensimetrics Corporation, Malden, MA, United States). Sound intensity was adjusted for each subject to be loud enough to be heard over the scanner noise. Stimulus delivery was controlled using Presentation software (Neurobehavioral Systems Inc., Albany, CA, USA).

Fig 1B shows an action trial. Each trial started with an auditory instruction, specifying the action to be executed (spoken words “ball”, “pen”, “slap”, “point”). The actors were instructed to mentally prepare their action during the 10 seconds that followed. Next, the LED lighted up indicating that the actor should perform the action once. For the object-directed actions, the actor was instructed to keep the hand on the object until the LED turned off (after 6 s). For the slapping and pointing actions the actor had to keep the palm (slap) or the side of the hand (pointing) on the table. When the LED turned off, the actor had to return the hand on the stomach. The execution phase was followed by an inter-trial interval (ITI) with duration of 12, 13, or 14 s, providing jittering to avoid subjects getting used to a specific ITI duration. The ITI durations were pseudorandomised and fixed across subjects to keep the data between subjects synchronized in time. The actors were instructed to keep their eyes on the LED at all times. The experiment comprised 5 runs with 24 trials in each, and the actors performed each action 6 times per each run. Order of actions was pseudo-randomized to control for possible order effects.

The hand actions were videotaped from a third person perspective with a HD camera positioned 5.5 m from the bore. The videos that were displayed in a subsequent fMRI experiment to the observer subjects were cut into 25-s segments that included a 10-s epoch before the action, 6 s of the action execution itself, and a 9-s ITI.

Seeing own actions can confound the experiment by providing similar visual input from the hand kinematics (yet with different viewpoints) to both actor and observer, which could subsequently drive the classifier performance. We therefore ran a control experiment with exactly the same setup with the exception that the actor kept her eyes closed throughout the whole experiment. This actor was one of the two actors who participated in the main experiment (female, age: 29). Action onsets and offsets were cued with sounds delivered via headphones.

### Experimental setup for observer subjects

In a subsequent fMRI experiment, twenty ‘observer’ subjects viewed the videotaped actions (Fig 1C). The observers practiced the execution of the actions before the experiment. Eleven observers viewed the stimulus videos of the first actor. Data from four additional observers of the second actor were collected. Because the results were essentially similar for the two different actor–observer subject groups, data were ultimately collapsed together. Finally, data for five additional observers for the closed-eyes actor were collected in the control experiment. A fixation cross was shown at the centre of the screen throughout the whole experiment, also during the period that separated the trials. The observers were instructed to watch the video and keep their eyes on the fixation cross. Each individual action observation trial started with a 25-s video (see the description above) and was followed by an ITI of 3–5 s. The ITI duration depended on the corresponding-trial ITI in actor’s experiment. The experimental structure was otherwise similar to that of the actor experiment (5 runs with 24 trials, 6 repetitions of each action per run). The videos were presented in the same order as the actions performed by the actor, using Presentation software (Neurobehavioral Systems Inc., Albany, CA, USA). Visual stimulation was back-projected on a semi-transparent screen using a 3-micromirror data projector (Christie X3, Christie Digital Systems Ltd., Mönchengladbach, Germany) and reflected via a mirror to the subject.

### Functional localizer tasks

Both actor and observer subjects performed two functional localizer tasks, one for action execution and another for action observation, at the beginning of the fMRI session. During the action execution localizer, the participants executed 28 power grip actions (see above) that started with an auditory cue followed by a 3-s pause. Next, a LED lit up for 3 s and the participants grabbed the ball and kept the hand on it until the LED turned off. ITI was randomized with possible values of 6, 7, or 8 s.

During the action-observation localizer, the participants viewed videos of actions similar to those used in the main experiment, recorded in the same setting but separately from the main experiment. Videos were presented in sixteen blocks with four videos in each. In additional twelve rest blocks, the participants viewed videos of a hand resting on the table. Each video lasted for 5 s and was followed by a 2 or 3 s pause. Breaks between blocks lasted for 9 s.

### fMRI acquisition and preprocessing

MRI scanning was performed with 3T Siemens Magnetom Skyra scanner at the Advanced Magnetic Imaging Centre, Aalto NeuroImaging, Aalto University, using a 20-channel Siemens head coil. Whole-brain functional images were collected using a whole brain T2*-weighted echo-planar imaging (EPI) sequence, sensitive to blood oxygenation level-dependent (BOLD) signal contrast, with the following parameters: 38 axial interleaved slices, TR = 2 s, TE = 24 ms, flip angle = 70°, voxel size = 3.1 x 3.1 x 3.0 mm, matrix size = 64 x 64 x 38. A total of 350 volumes were acquired in each run, and the first 4 volumes of each run were discarded. High-resolution anatomical images with isotropic 1 x 1 x 1 mm voxel size were collected using a T1-weighted MP-RAGE sequence.

FMRI data were preprocessed using MATLAB (The MathWorks, Inc., Natick, Massachusetts, USA) and FSL (FMRIB's Software Library, www.fmrib.ox.ac.uk/fsl). After slice timing correction, the functional images were realigned to the middle scan by rigid-body transformations with MCFLIRT to correct for subject motion. Next, non-brain matter from functional and anatomical images was removed using Brain Extraction Tool (BET); [21]. Functional images were registered to the MNI152 standard-space template (Montreal Neurological Institute) with 2-mm isotropic voxel resolution to simplify the analysis pipeline. The transformation parameters were acquired by first calculating transformations from structural to standard space and from functional to structural space, and then concatenating these parameters. Next, these transformation parameters were used to co-register functional datasets to the standard space. Both registration steps were performed using FLIRT [22]. Motion artefacts were cleaned from the functional data using 24 motion-related regressors [23], signal from white matter, ventricles and cerebro-spinal fluid were also cleaned from the data. While this approach is more conservative than the more traditional 6 motion-parameters regression, we chose it because the motor task our subjects performed in the scanner potentially increased the amount of head motion. This decision was done *a priori* and no other motion-correction strategies were implemented.

For classification analyses, the data were first down-sampled to 4-mm isotropic voxels because some of the employed classification analyses were computationally prohibitive. For the sake of consistency, all reported classification analyses, including within-subject classification, were done on the down-sampled data. Spatial smoothing was applied to the non-downsampled data as the final preprocessing step only for the analysis with the general linear model (GLM), with a Gaussian kernel of FWHM 8 mm.

### Univariate analysis

Task-related responses to action execution (actors) and observation (observers) were analysed using the two-stage random effects analysis with GLM implemented in SPM12 (www.fil.ion.ucl.ac.uk/spm). Four regressors (power grip, precision grip, slap and pointing) were used to model fMRI voxel time series. Boxcar function was used to model BOLD responses; it included only the time points during which the action was viewed or executed (the trial duration was 6 s, equalling 3 samples); thus the model did not include the preparation phase. Regressors were convolved with the canonical hemodynamic response function to account for hemodynamic lag. The first level model in SPM included high-pass filter with 256-s cut-off. After generating individual contrast images for each action, a second level (random effects) analysis was applied to these contrast images for observer subjects (N = 15) using a t-test in SPM12. Statistical threshold was set at *p* < 0.05, false discovery rate (FDR) cluster corrected. The data of actors (N = 2) was summarized by averaging across both subjects.

In the control experiment with the closed-eyes actor, the univariate analyses were performed in the same way as described above to compare the activated brain regions when the actor was seeing versus not seeing own hand movements. The statistical image for closed-eyes actor included the GLM results from the first-level model in SPM (N = 1), and the statistical image for the observers of the closed-eyes actor included the results of the second-level model in SPM (N = 5).

Localizer tasks were analysed using GLM, where for each individual, two contrast images were generated: main effect of action execution from action-execution localizer, and action observation *versus* observation of actor resting (not performing any actions) contrast from action-observation localizer. Subject-wise t-statistic maps (N = 15) were subsequently used to generate individual regions of interest (ROIs) for pattern classification analyses. The maps were thresholded at T > 2 as a feature selection step, and subsequently binarized. Liberal threshold was chosen as these images were not used for statistical inference, but rather as feature-selection filters.

### Region-of-interest selection

First, we derived the regions for two distributed ROI masks from the subject-wise action execution and action observation localizers for each individual subject. Here we use the term ROI to refer to the spatially distributed set of voxels (not necessarily adjacent) used as a single mask in classification analysis. Then we created an overlap localizer distributed ROI, which was similar for all the subjects, by combining the voxels in individual action execution and action observation ROIs thresholded at T > 2 (see Table 1). These data-driven distributed ROIs are well suited for controlling individual variability in action execution and observation across the subjects of our study.

**Table 1:** MNI coordinates of clusters included in the overlap localizer ROI. The individual subject data obtained in action-execution and action-observation localizers were thresholded at *T* > 2, and supra-threshold voxels overlapping in both execution and observation were preserved. Labels provided from Harvard-Oxford cortical and subcortical structural atlas (FSL).

Region	Hemisphere	x	y	z	size
Lateral occipital cortex	L	–50	–76	–12	712
Lateral occipital cortex	R	52	–72	–6	788
Lateral occipital cortex, superior division	R	26	–86	18	274
Supramarginal gyrus	R	60	–44	20	170
Cuneal cortex	L	–12	–90	22	116
Supramarginal gyrus	L	–54	–28	34	1122
Postcentral gyrus	R	30	–36	50	556
Precentral gyrus	L	–24	–10	54	203
Precentral gyrus	R	28	–10	58	64

However, our localizer results did not include some of the regions implicated in the motor mirror circuitry (e.g. inferior frontal gyrus (IFG); [24–26]). Thus, in a second approach we generated a distributed meta-analytic ROI consisting of activation foci corresponding to studies with keyword “grasp” in the Neurosynth.org database and combined forward and reverse inference maps (date of acquisition: 11.10.2013; [27]). Because the role of the anatomically defined Broca’s region in human mirror-neuron system is still under discussion (for review, see [28, 29]), we included a meta-analytic distributed ROI that comprised the multiple regions included in IFG (e.g. BA44, BA45 and BA47). While these regions differ in their functional roles, we did not have a prior hypothesis on the role of the subregions in sharing action-related brain activation signatures, and therefore we included all three subregions as a single ROI as this was, to our knowledge, the first-ever hyperclassification investigation on action observation and execution. The resulting image with meta-analytic foci was subsequently binarized and used as a distributed ROI comprising several distinct nodes, such as the bilateral LOC, SPL, right SMG, precentral and postcentral gyri and inferior frontal cortex (see Table 2).

**Table 2:** MNI coordinates of clusters included in the meta-analytic ROI. Labels provided from Harvard-Oxford cortical and subcortical structural atlas (FSL).

Region	Hemisphere	X	y	z	size
Temporal occipital fusiform cortex	R	38	–48	–24	858
Temporal occipital fusiform cortex	L	–44	–52	–22	725
Gyrus rectus	R	0	0	–6	110
Inferior frontal gyrus	L	–50	4	6	3885
Inferior frontal gyrus	R	56	10	12	159
Supramarginal gyrus	R	58	–38	14	1445
Lateral occipital cortex	L	–54	–68	24	79
Paracingulate gyrus	L	–8	8	42	304
Postcentral gyrus	R	38	–24	50	170

To control for possible low-level visual confounds in the classification (resulting from actor subjects seeing their own hand movements, leading to similar kinematics of seen activation in action and observation conditions), the primary visual cortex (V1) was excluded from all the distributed functional ROIs. Furthermore, the V1, V2 and V3 regions combined were used as a separate ROI to investigate predictive accuracy of low-level visual areas. The anatomical locations of V1, V2 and V3 were derived from the Jülich Histological Atlas in FSL [30]. To provide additional control for influence of visual information from the observation of one’s own movements in the actors we created an additional distributed ROI consisting of a cluster spanning LOC and EBA (5-mm spheres centered at 50–64 4, and –48–70 4). Finally, since the action-specific information is supported by the premotor cortex [3, 13], we also included a control ROI comprising only this region (defined by the Jülich Histological atlas in FSL; [30]).

### Pattern classification

Pattern classification was performed in three ways. First, we wanted to establish that each of the executed and observed actions would be associated with distinct brain activation signatures. To that end we performed a conventional within-subject classification on all subjects, including actors and observers, by training and testing the classifier on single subject data. Second, to test whether action execution and observation would be associated with similar brain activation signatures in actor’s and observer’s brains, we initially performed between-subjects classification without functional realignment. In this approach, the individual actor’s data were used to train the pattern classifier to distinguish between the four different actions, and the classifier was tested using data from the observer who saw the movements executed by that actor. Third, to test whether the neural codes for action observation and execution would contain similar action-related information, that is misaligned between the actors and the observers, we performed hyperclassification analysis where an additional functional realignment step was employed before the classification.

For all tested classifiers the input data comprised all trials with 3 scans per trial recorded during action execution or observation phases of the experiment, and shifted by 6 s to account for the hemodynamic lag. We did not use temporal compression approaches, such as fitting a per-trial GLM to use beta maps as training/testing inputs. Instead, we analyzed the preprocessed data as they were. While our approach could be considered more conservative than using per-trial GLM due to inclusion of noisier data, it provided more examples of training data, resulting in a potentially more robust classifier. Each scan was used as an independent training or testing example. We evaluated the performance of the classification models in all three classification approaches using leave-one-run-out cross-validation framework, where four runs were used to train the classifier and the left-out run was used in testing, and the process was repeated iteratively for each run. In total, 360 samples were used per subject (within-subject analysis) or 720 samples per subject pair (between-subjects analysis and hyperclassification), i.e. 3 samples per trial, 24 trials per run and 5 runs per subject. A training set in each iteration of cross validation included 288 samples, and testing sample included 72 samples. In within-subject classification analysis the training and testing data were taken from a single subject. In hyperclassification and between-subjects analyses the classifier model was trained on the runs taken from the actor's data, and the testing runs were taken from the observer's data.

The significance of the mean classification accuracies was tested by comparing their 95% confidence intervals to the theoretical chance level. Since empirical chance level accuracy can differ from theoretical chance level [31], we verified it using 100 random permutations of the class labels. The subject-wise, between-subjects classification and hyperclassification accuracies were approximately normally distributed; hence the confidence intervals for their means were obtained from Student's t-distributions.

Classification was accomplished with Bayesian logistic regression with a sparsity promoting Laplace prior (see [32, 33] for mathematical description of prior). Each individual voxel weight within a ROI was given a univariate Laplace prior distribution with a scale hyperparameter, which was optimized separately for each subject or subject pair by maximizing the average accuracy over all other subjects or subject pairs ([34]; candidate values 0.01, 0.04, 0.21, 1, 4.64, 21.54, 100). The multivariate posterior distribution of classifier weights was approximated using the expectation propagation algorithm [33] implemented in the FieldTrip toolbox [35]. Four binary classifiers were trained to discriminate between each action category versus the others combined. The training data thus included 72 samples of the target class and 216 samples of all other classes. The classification performance was tested by collecting the class probabilities for each pattern in the testing set using the binary classifiers, and assigning the class with the maximum probability to each pattern.

### Functional realignment and hyperclassification between two brains

As stated above, our hyperclassification approach aimed at classifying an observer’s brain activation on the basis of the actor’s brain activation. To account for differences in functional localization of action generation and observation across individuals, an additional functional realignment step was introduced in the analysis pipeline. We used Bayesian canonical correlation analysis (BCCA; see [36] for detailed mathematical description) to perform the realignment step prior to hyperclassification. Realignment was performed on the unlabeled data. BCCA was implemented using R CCAGFA package [36, 37]. The BCCA—with actor-specific, observer-specific, and shared components—models the structured variation (covariance) in the brain activities of the two interacting subjects (the individual who executes an action and the individual who observes it), with three types of components: actor-specific, observer-specific, and shared. The model automatically assigns the components to one of the three types via a group-wise sparse automatic relevance determination prior [36]. The shared components provide a linear transformation between the actor's and observer's brain-activity spaces. Given the brain activity of an observer, the linear transformation (realignment) is used to predict what this activity would look like in the actor's space. The modality-specific components are used to explain away actor- and observer-specific structured variation, which helps the estimation of the shared components [36]. A relatively small number of components (low-rank transformation) are used to avoid overfitting.

**Fig 2:**
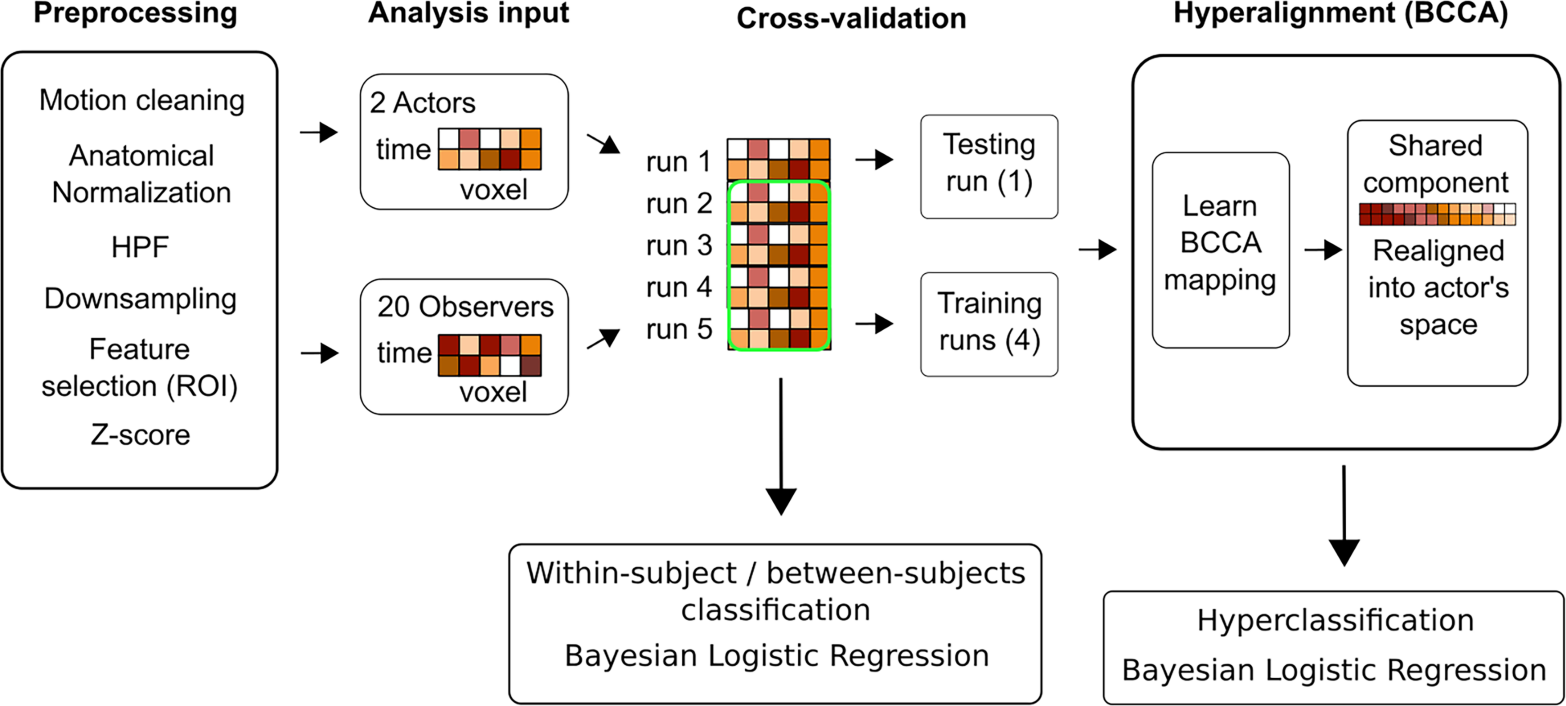
Schematic description of data preprocessing and analysis for hyperclassification. Bayesian canonical correlation was used on preprocessed data to acquire mapping between actor’s and observer’s BOLD signals. Mapping was acquired in cross-validated fashion, where a model was trained on four runs of the actor and the observer. The observer’s left-out run was used in subsequent analysis, where shared representation between actor and observer was mapped to actor’s functional space and used in testing the classifier. Bayesian logistic regression was used as pattern classifier. In within-subject classification training and testing was done using the data from the same individual. In hyperclassification training was done on actor’s data and testing on corresponding observer’s data.

The setting for training and testing the hyperclassification was similar to and compatible with the within-subject classification. First, for each actor–observer pair, the number of components estimated was optimized (candidate values 20, 30, 40, 50, 60, 70, 80, 90, and 100) simultaneously with scale hyperparameter for classifier, by maximizing the average classification accuracy over all other subjects. Then, the data of the current actor and observer were separated into training and testing sets for the cross-validation, where four runs from the actor and the observer were used in training the BCCA model (given the number of components), and one left-out run from the observer was used to generate the realigned data. Subsequently, the classifier was trained only on the actor's data from the four runs (given the scale hyperparameter) and tested on the functionally realigned observer's run (Fig 2).

### Characterizing the data after functional realignment

If functional realignment allowed successful hyperclassification, the next question would be i) where in the brain the similarity between the actor's and the observer's neural activation increased through functional realignment and ii) whether a local increase in similarity leads to corresponding local increase in classification accuracy. To this end, we first calculated intersubject correlations (ISC; [38]) between the brains of actors and observers before and after realignment (N of pairs = 15), assuming that successful realignment would increase ISC of voxel-wise time series in brain regions where shared information between actors and observers increased. Because during functional realignment we allowed remapping of voxel activation to any place within a ROI, this step allowed us to investigate whether remapping would be specific to some regions within a ROI or randomly distributed across the ROI. In the latter case the realignment model would be theoretically meaningless as correlation would increase and decrease randomly across the brain.

Pearson correlation coefficient r was used to characterize the strength of the ISC for each voxel for each actor–observer pair before and after the realignment. The data for a single subject included all samples, e.g. 3 scans per action, 24 actions per run, 5 runs, totalling 360 scans per subject. First, for each actor and corresponding observer, a single r value was computed for each voxel using the data without functional realignment, resulting in a single voxel-wise r-statistic map per actor–observer pair (N = 15). Then, similar computation was done on each actor–observer pair with observer’s data functionally realigned, similarly resulting in a single r-statistic map per pair. These r-statistic maps of intersubject correlations before and after the functional realignment were Fisher-transformed and compared with each other using two-sample t-test (N = 30; 15 with functional realignment vs 15 without functional realignment) to reveal brain regions that became statistically significantly more similar after the realignment. The resulting T-statistic map was FDR-corrected at *q* < 0.05 [39] and thresholded with cluster size of 125 voxels to enhance visualization.

The ISC analysis reveals only the regions where similarity between actors and observers increased after functional realignment, but does not provide information on whether these regions were relevant for action hyperclassification. To reveal brain regions where realignment would increase hyperclassification accuracy, we used a k-nearest-neighbour (kNN) classifier [40, 41] based on spatiotemporal ISC matrices across all voxels within a spherical searchlight. Searchlight kNN analysis extends the ISC analysis by revealing brain areas where hyperclassification accuracy increased significantly after the realignment. A single searchlight contained 19 voxels (6 mm^3^). For each actor–observer pair and for each searchlight and trial, a single Pearson correlation value was computed across three scans corresponding to one trial and across all voxels within the searchlight (n = 19x3 = 57 data points). Thus, with 24 trials in a single run, and with 5 runs altogether, the correlation matrix for one actor–observer pair had dimensions of 120 by 120, where each cell corresponded to the correlation value for a trial between actor and observer.

The classification was performed by taking ISC data for each trial (column in correlation matrix) and assigning to this trial the same class (power grip, precision grip, slap or point) as assigned to the majority of its most similar neighboring trials, where the number of evaluated neighbors corresponded to the k-value. The analysis was performed separately for k-values ranging from 1 to 120 with a step of 6. We used mean classification accuracy over all k-values to control for possible sensitivity of kNN classifiers to noise at low k-values [42]. After the analysis was done for each searchlight before and after the functional realignment, the difference was tested using permutation-based t-test. Statistical threshold was set at *q* < 0.05, FDR-corrected [39].

### Validation of BCCA and hyperclassification

Three validation approaches were used to ensure that realignment achieved with BCCA and the subsequent improvement of classifier performance do not reflect merely realignment of the noise present in the data:

1. **Classifying the temporally misaligned observer's and actors’ data.** First, we shuffled the observer’s data in time, and subsequently performed functional realignment and hyperclassification on these data to verify that this procedure leads to chance-level accuracy. If hyperclassification accuracy stays above chance level for temporally misaligned data, we can conclude that the BCCA model is realigning task-independent noise across the data sets.
2. **Realigning and classifying simulated surrogate data.** We next trained and tested the classifier with BCCA-aligned random noise filtered with BOLD spectra that were acquired from actual observers’ data recorded during the experiment. Separate datasets were simulated for each observer. Actor’s data were used in training the classifier, and surrogate observer’s data were used in testing. While surrogate data retain the characteristics of real BOLD signals, they lack the temporal structure of the actual experiment. In case the BCCA approach would just match noise and real data, this analysis should provide above-chance classification accuracy. If hyperclassification with simulated surrogate data is unsuccessful, we can conclude that what is realigned is more than mere noise.
3. **Realigning and testing classification in control ROI data**. Finally, if functional realignment allowed successful classification in a region unrelated to action observation and execution, the model would not be robust against noise as it would generate meaningful signal where there is none. According to previous literature, frontal pole, cingulate cortex and temporal poles are not directly involved in action execution or observation [43], and therefore functional remapping between these regions should not give above-chance-level accuracy for hyperclassification. Such task-unrelated ROIs were thus used to test whether the BCCA model could erroneously generate data that look similar for actors and observers, while the brain signals used for model training didn’t have the shared action-related components. Consequently, ROIs for these regions were generated using the Jülich Histological Atlas in FSL [30], and functional realignment and classification were then attempted for this set of ROIs.

## Results

### Task-related BOLD responses

**Fig 3:**
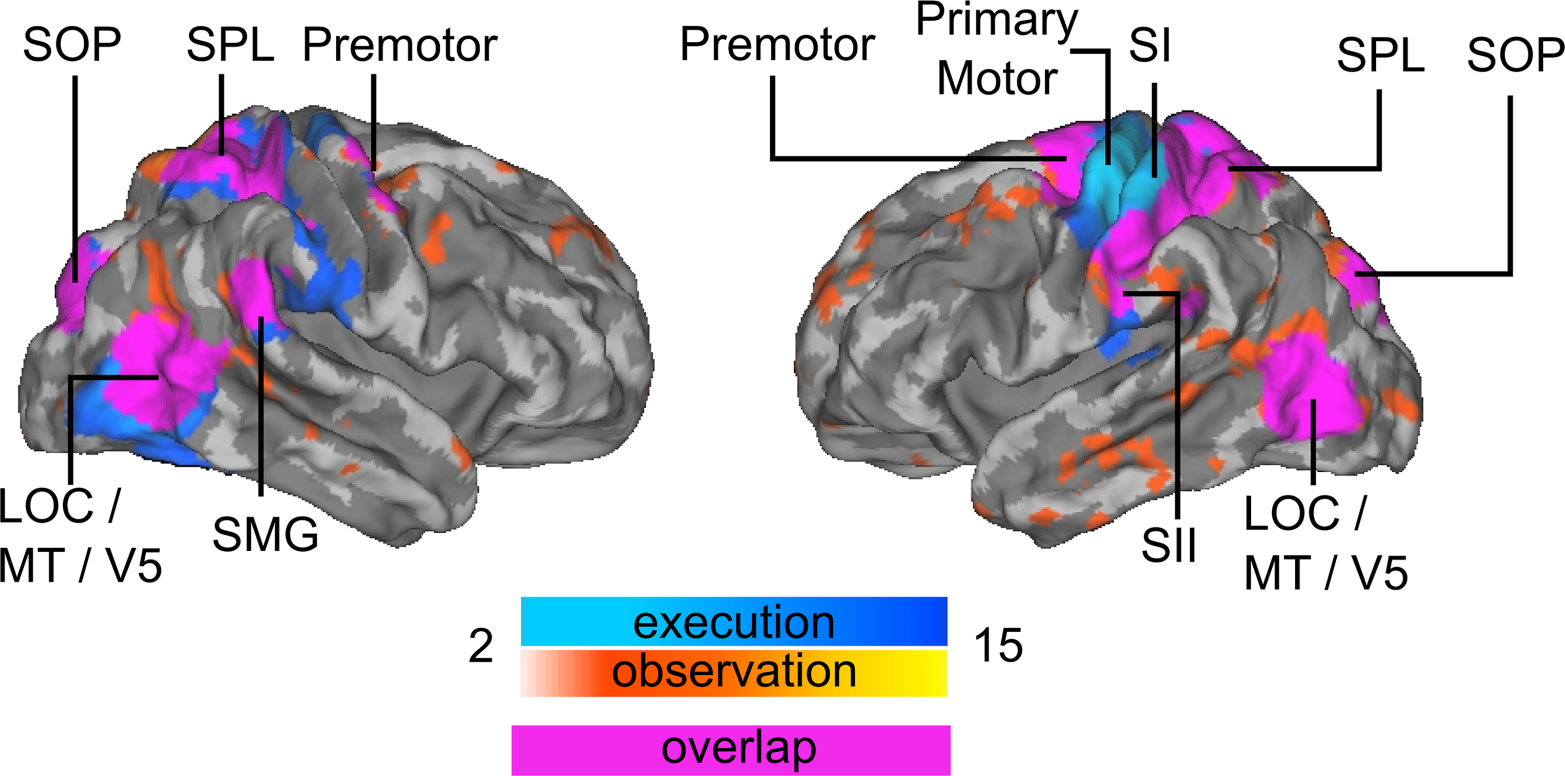
Brain regions showing activation during action execution and observation localizers. For each subject, the statistical image was thresholded at T > 2 and binarized. Warm colors indicate number of subjects that showed activation during action observation for each voxel, and cold colors during action execution. Purple color shows overlap between action execution and observation. Abbreviations: SPL–Superior Parietal Lobule; SOP–Superior Occipital Pole; SMG–Supramarginal Gyrus; LOC–Lateral Occipital Cortex, SI–primary somatosensory cortex, SII–secondary somatosensory cortex.

Fig 3 shows cortical areas activated during localizer tasks (N = 15 observer subjects). Activations for action execution and action observation localizers overlapped in a cluster spanning bilateral dorsal LOC (cluster also included MT/V5), also extending to extrastriate body area (EBA), SMG, SPL, and secondary somatosensory cortex. No overlap was observed in cerebellum. For action execution localizer, neural activity was observed for multiple subjects in the superior part of the left precentral cortex, left primary and secondary somatosensory cortices, right cerebellum, a cluster spanning LOC, V5 and EBA, supramarginal gyrus (SMG), premotor cortex and superior parietal lobe (SPL). During action observation localizer, neural activity was observed also in bilateral cluster spanning LOC, V5 and EBA, SMG, SPL and premotor cortex. Only the sum of binarized single-subject statistical maps is shown (Fig 3), as these ROIs were used in the hyperclassification that requires similarly sized ROIs across participants.

**Fig 4:**
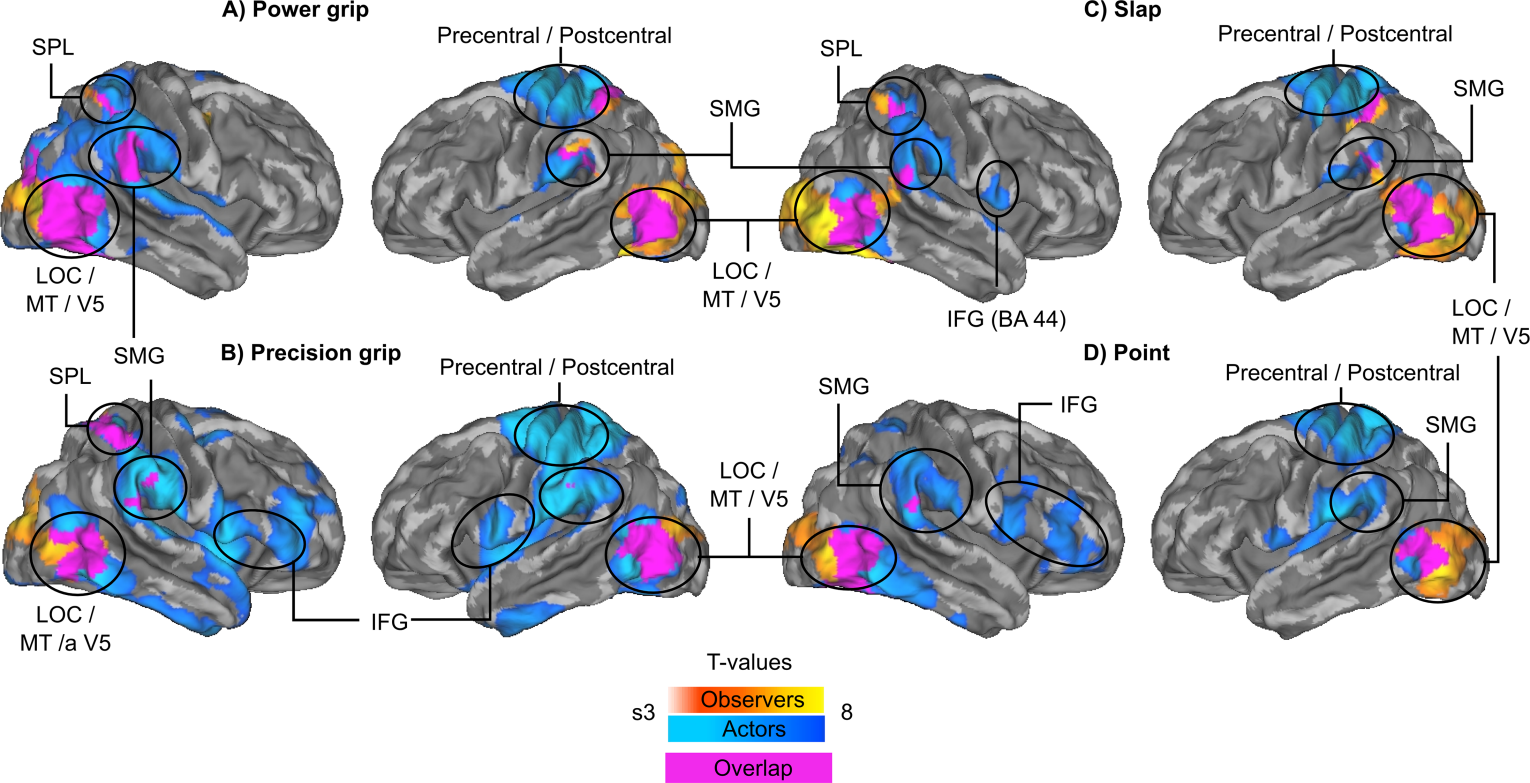
Action-specific brain activation. Brain regions whose activity increased during specific actions (A: Power grip, B: Precision grip, C: Slap, D: Point) during the main experiment. Warm colors indicate activation for observer subjects, cold colors activation for actor subjects, and purple color overlap between the activation maps of actors and observers.

**Fig 5:**
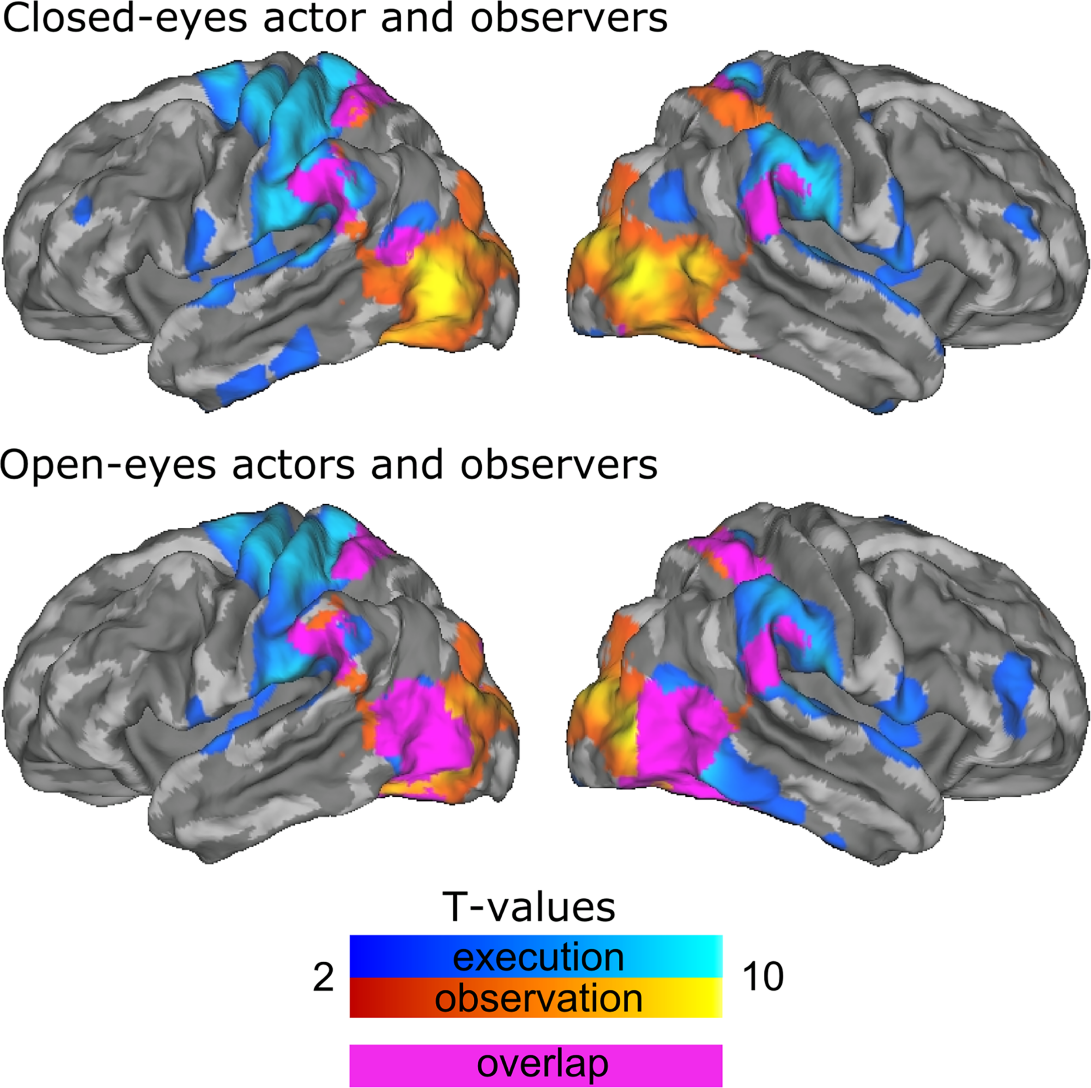
Brain activation during closed-eyes and open-eyes experiments. Brain regions showing significant activation during action execution and observation in the main experiment for closed-eyes (top) and open-eyes (bottom) conditions for actor and all observers. Warm colors indicate activation for observer subjects; cold colors indicate activation for actor subjects. Purple color indicates overlap between activation maps of actors and observers.

Several regions were activated during execution of all action categories in the main experiment (Fig 4, cold colours, N = 2 actor subjects), specifically left precentral and postcentral cortices, right cerebellum and left cerebellar VI, bilateral SMG and bilateral dorsal LOC together with V5. Action observation (Fig 4, warm colours, N = 15 observer subjects) of all different actions elicited remarkably similar neural activations in bilateral LOC extending to lingual gyrus and intracalcarine cortex and right SMG. Observing power grip and slap also activated left SMG and bilateral SP, whereas observing precision grip also activated right SPL. Execution of specific action categories activated some additional areas. During precision grip and point actions, also large portions of bilateral inferior frontal cortex were activated. Slapping activated pars opercularis of right IFG. GLM analysis of the closed-eyes actor and corresponding observers (N = 5) revealed that regions involved in action execution in closed-eyes actor did not include LOC (Fig 5).

### Within-subject action-execution and action-observation classification

**Fig 6:**
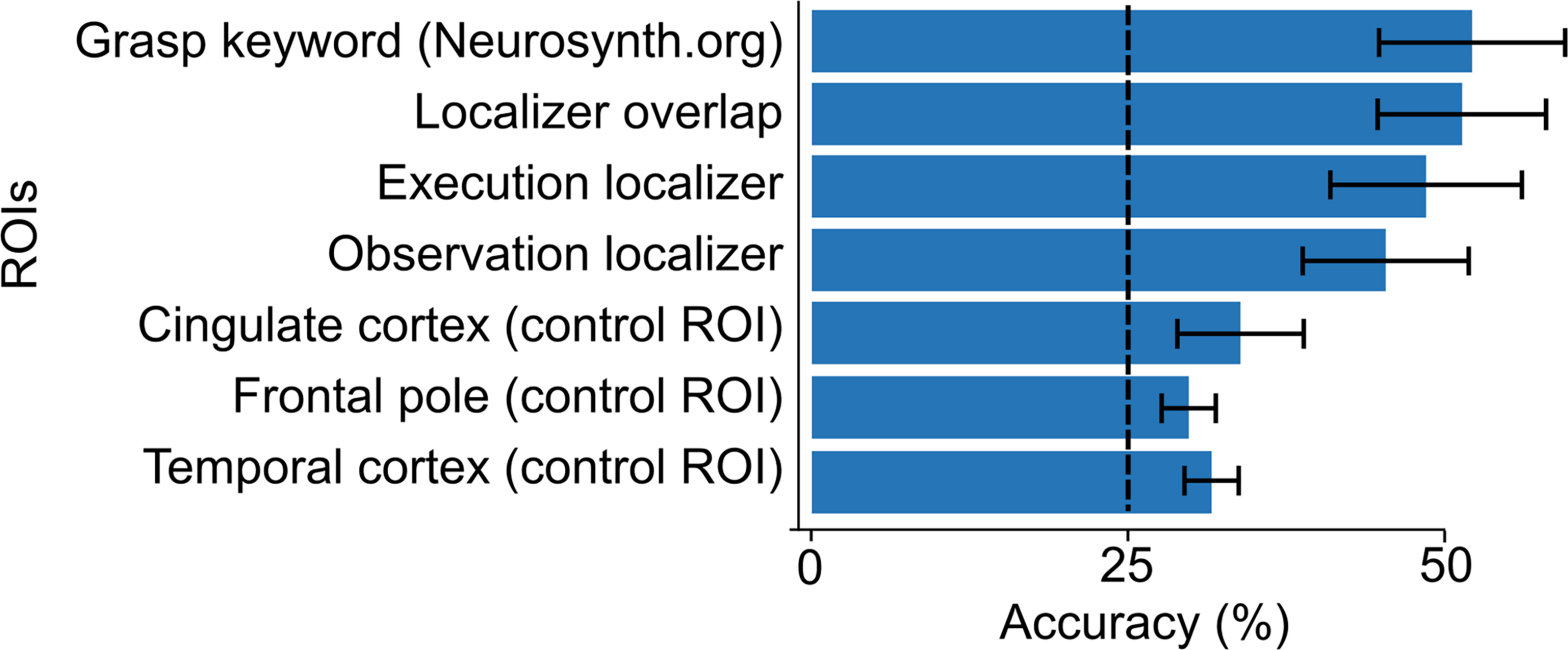
Within-subject classification accuracies. Means and 95% confidence intervals for within-subject classification of seen actions in different regions of interest (ROIs). Dashed line indicates the chance level.

We initially ran conventional within-subject pattern classification analyses to test whether it is possible to differentiate between different executed or observed actions in the first place. For within-subject action execution classifier (N = 2 actor subjects), both localizer and meta-analytic ROIs yielded accuracies statistically significantly above chance level of 25%. Mean accuracy over subjects was 65% for action execution localizer ROI, 59% for action observation localizer ROI, 66% for overlap localizer and 70% for meta-analytic ROI (95% CIs: 63–67%, 30–87%, 48–85% and 48–85%, respectively). Within-subject classifier for action observation (N = 20 observer subjects) provided above chance level accuracy in all ROIs, ranging from 44% to 51%. Accuracy was highest (51%) for meta-analytic ROI, followed by overlap (50%), action execution (47%) and action observation (44%) localizer ROIs, although these differences were not statistically significant (95% CIs: 39–55%, 37–51%, 44–57% and 44–59%, respectively; Fig 6). Accuracies in the visual cortex ROIs (V1 and V1+V2+V3) were 57% and 59%, respectively.

### Brain-to-brain hyperclassification

**Fig 7:**
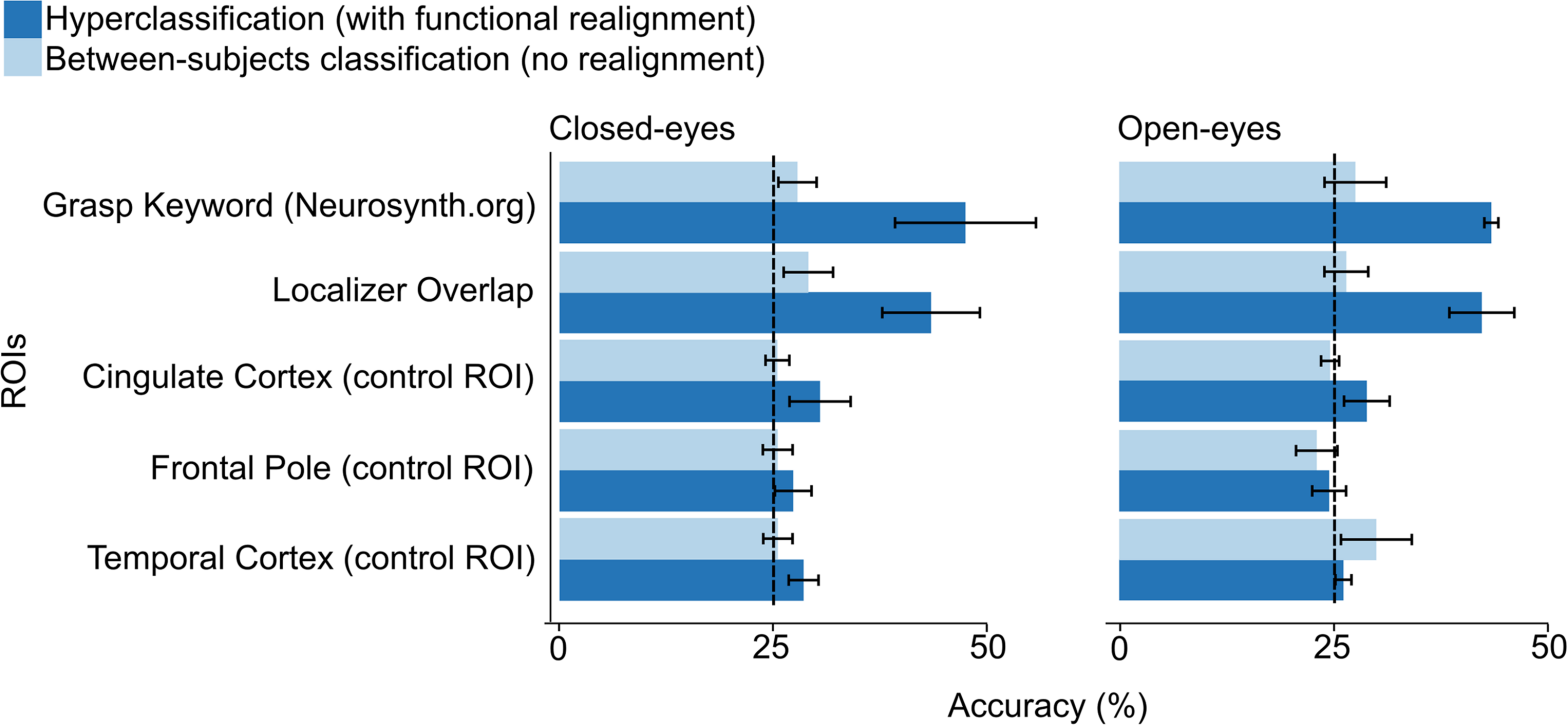
Hyperclassification and between-subject classification accuracies. Means and 95% confidence intervals for hyperclassification and between-subjects classification accuracy of seen actions in different regions of interest (ROIs) in the closed-eyes (left) and open-eyes (right) conditions. Dashed line indicates the chance level.

Without functional realignment, mean between-subjects classification accuracy was only slightly higher than chance level in the meta-analytic and action execution and action observation localizer overlap ROIs (25% chance level; 95% CIs: 26–29% and 26–30% respectively; Fig 7). Accuracies in the visual cortex ROIs (V1 = 25%; V1+V2+V3 = 26%) did not differ statistically significantly from chance level.

**Fig 8:**
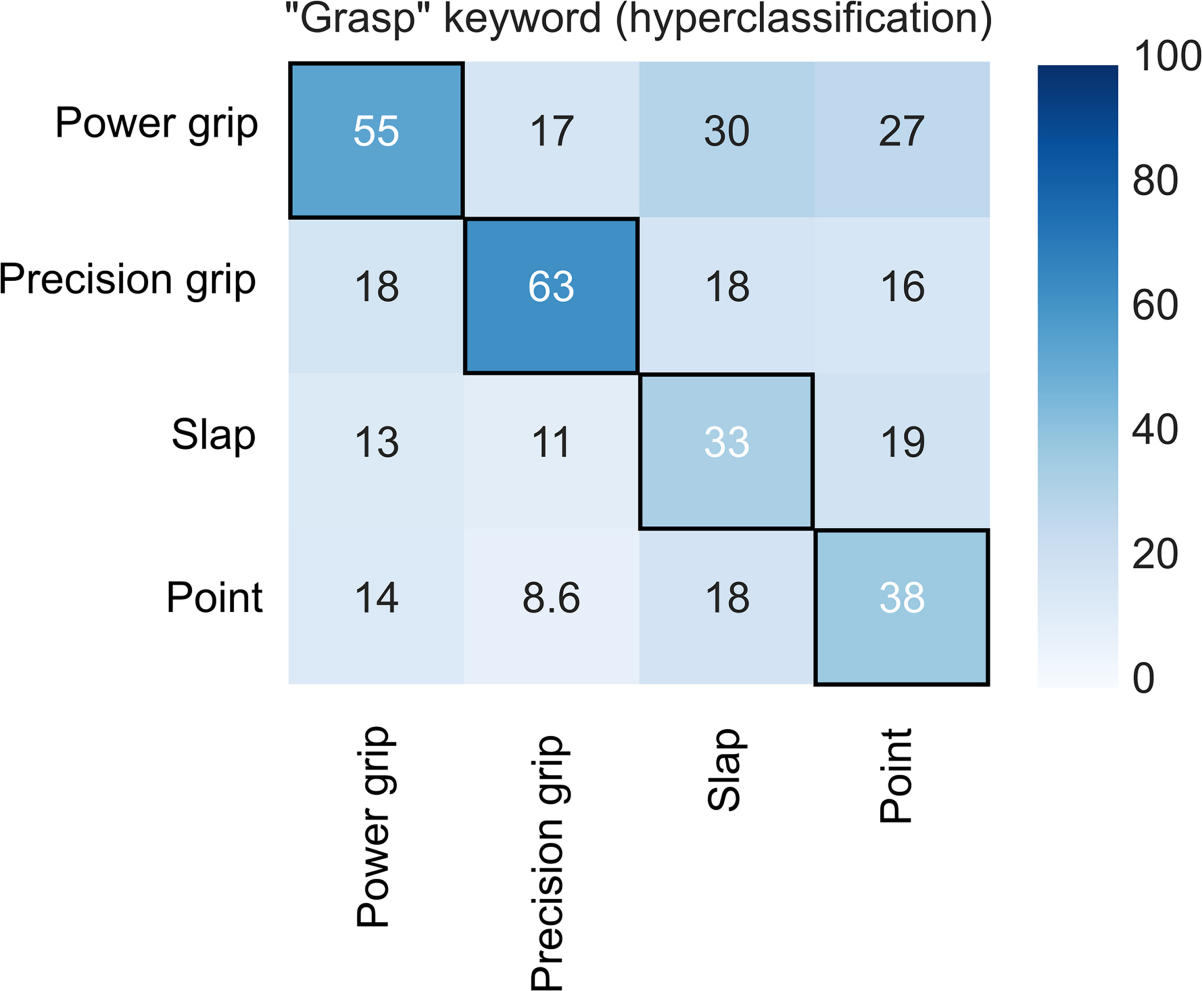
Confusion matrix for functionally realigned hyperclassification with meta-analytic ROI. Numbers in the cells indicate percentage of action category guesses by classifier. Black frames denote action categories that were correctly classified with above chance level accuracy.

After functional realignment, mean hyperclassification accuracy exceeded chance level statistically significantly in both ROIs (25% chance level; 47% for meta-analytic and 44% for localizer, CIs: 40–56% and 38–49% respectively; Fig 7, confusion matrix for meta-analytic ROI: Fig 8), and increased in comparison to between-subjects classification (meta-analytic ROI: T = 7.7, p < 0.001, mean increase = 19 percentage points; localizer overlap: T = 6.7, p < 0.001, mean increase = 16 percentage points). Hyperclassification accuracy following functional realignment did not differ statistically significantly between the meta-analytic and localizer ROIs. Accuracy in the visual-cortex ROI (V1+V2+V3) was 50%. Hyperclassification analyses with premotor cortex ROI yielded mean accuracy of 32%, CI: 28–35%. The EBA / LOC ROI yielded mean accuracy of 32%, CI: 30–35%.

Absence of temporal gaps between preparation and execution phases of the trial for actors could have caused signal from action planning to leak into the execution signal, thereby creating a potential confound because action imagery and action planning recruit similar brain regions as does action observation [44]. To control for this confound, we reanalyzed the data after shifting all actors’ events ahead by 2 TRs (4 s). While the accuracy decreased, it still remained above chance level, being on average 49% (vs 64%) for meta-analytic ROI in actors, and 36% (vs 46%) for meta-analytic ROI in hyperclassification analysis.

Mean hyperclassification accuracy of a model trained on the closed-eyes actor and tested on five observers significantly exceeded chance level of 25% in both meta-analytic (43%, CI: 42–44%) and action execution and action observation localizer overlap ROIs (42%, CI: 38–46%; Fig 7).

**Fig 9:**
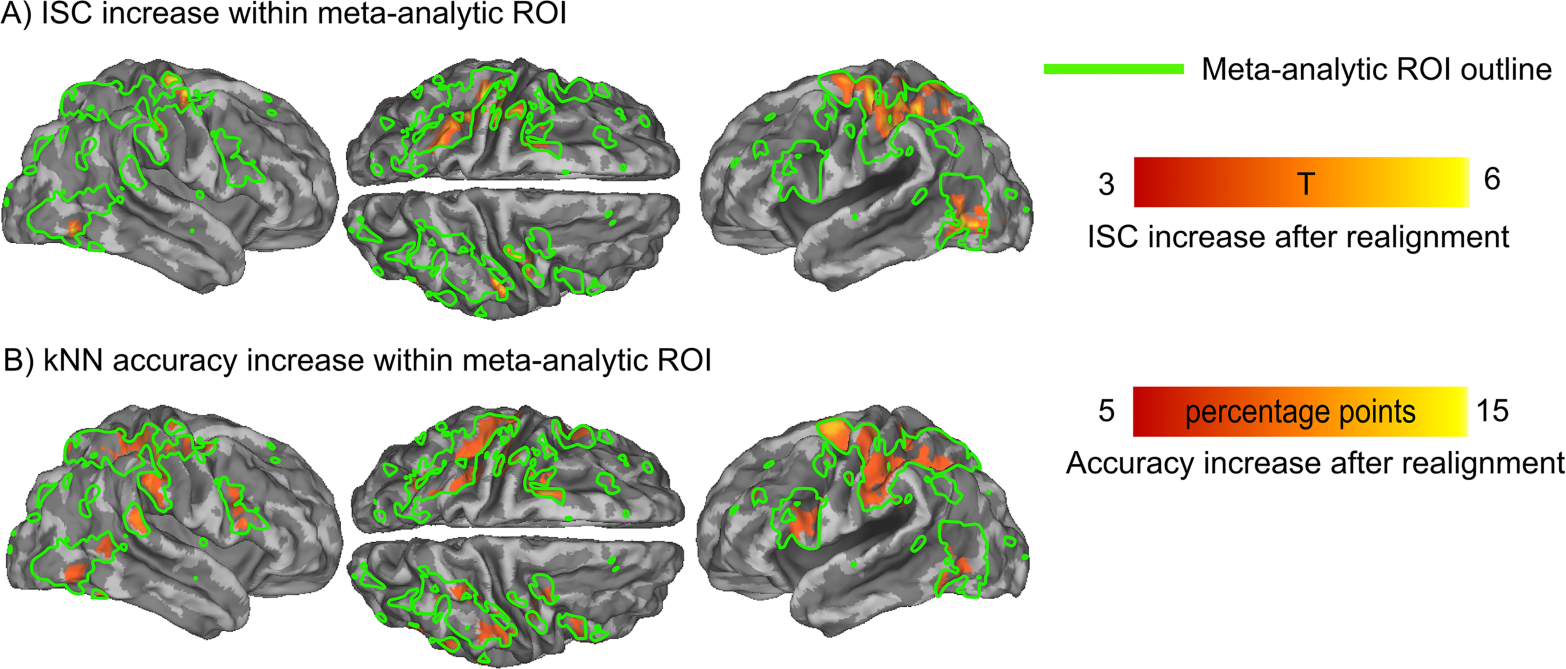
Increases in intersubject correlation and classification accuracy after functional realignment. A) Cortical regions showing significant increase of intersubject correlation (ISC) between actors and observers after functional realignment. Colorbar denotes the difference in ISC, indicated with T-statistic. Green outline indicates the regions included in meta-analytic ROI. B) Cortical regions showing significant increase in searchlight kNN classification accuracy after functional realignment thresholded with increase of accuracy of more than 5 percentage points. Colorbar denotes the difference of kNN accuracy. Green outline as in A).

Next, we calculated voxel-wise intersubject correlation within the meta-analytic ROI between actor–observer pairs before and after the realignment to reveal the regions whose similarity between the actor and the observers increased following functional realignment. The meta-analytic ROI was used since it covers a larger number of brain regions potentially involved in action execution and observation than the localizer overlap ROI. Statistically significant increases were observed in bilateral LOC, SMA, and more profoundly in left SPL and premotor cortex (q < 0.05, FDR-corrected; Fig 9A). KNN searchlight classifier in the meta-analytic ROI revealed that functional realignment increased classification accuracy statistically significantly in all searchlights within the ROI, with premotor cortex, right pSTS, bilateral SPL, bilateral pars opercularis (BA 44 of IFG) and LOC showing more than 5 percentage points increase (Fig 9B).

Finally, all validation approaches confirmed that the functional realignment works on task-related signal rather than noise. When temporal alignment between the data of the observer and the actor was broken by shuffling or shifting the observer’s data time points, the realigned data could no longer be accurately classified using the hyperclassification approach (accuracy for each ROI remained at the chance level of 25%). Realigning the simulated data while keeping the noise structure, revealed that no artificial temporal structure was introduced to the data, as classification accuracy for each tested ROI remained at the chance level of 25%. Training and testing the classifier with functionally unrelated ROIs in cingulate cortex, frontal pole and temporal cortex yielded accuracies only slightly exceeding chance level and significantly below any other ROI (means: 30%, 25% and 28% respectively; 95% CIs: 27–33%, 23–28% and 27–30% respectively, Fig 7).

All validation approaches with the closed-eyes subject revealed similar results as the main experiment. Specifically, temporal misalignment and testing with surrogate data resulted in chance-level accuracy, and unrelated regions also showed low accuracies (cingulate cortex: 27%, frontal pole: 24%, temporal cortex: 28%).

## Discussion

We showed that the action-specific neural activation patterns associated with observing and executing different actions share enough similarity to allow successful brain-to-brain hyperclassification between individuals executing and observing actions. Both execution and observation of actions were associated with action-specific brain activation signatures. Fine-scale patterns for executing and observing an action differed across individuals, and thus functional alignment was required to match these patterns between the actor and the observers. These results provide support for the common-coding hypothesis of action observation and execution [45, 46] and reveal how the shared brain activation signatures between action execution and observation can be extracted and used to map (a part of) the brain state of the observer to that of the actor. We found that both action execution and observation were supported by activity in an extensive brain network beyond the core mirroring systems, in agreement with previous findings [47, 48].

### Hyperclassification requires functional realignment to reveal shared neural codes

We addressed the “misalignment” of brain activation signatures between two tasks (execution and observation) and two individuals (actor and observer). Such misalignment can have multiple possible reasons. First, the brain activation signatures for action execution and observation can be misaligned already in a single individual. However, previous research rather suggests that action execution and observation in single individuals are associated with similar, distributed cross-modal neural activity patterns [14]. Second, the misalignment could be due to inter-individual differences. For example, the activity of a single ROI could allow successful classification of a certain action separately from the actor and observer, but still these activity patterns could be misaligned so that one individual’s action-execution brain activation signature (that allows classification of the action) would not be the same as the similarly located activation signature for action observation in another individual. The present findings address misalignment in the latter case where neural activity patterns related to action execution and observation in two individuals contain similar information, yet are misaligned across the individuals.

While it is non-trivial to visualize the misalignment, results of ISC and kNN analyses show brain regions where spatiotemporal patterns of neural responses became more similar and more informative between actors and observers after the realignment. Specifically, actor–observer ISC analysis revealed that functional realignment increased the similarity of voxelwise time series in bilateral LOC, left SPL and premotor cortex. However, because ISC only reflects similarity of the time series of BOLD signal, ISC increases could occur in areas where no action-related information is available, and we therefore also tested which voxels contained action-sensitive information. Increase of spatiotemporal action-specific information was revealed by increased searchlight classification accuracy following functional realignment, notably in bilateral premotor cortex, right SMG, bilateral LOC, bilateral pars opercularis and right pSTS that all showed over 5 percentage point increase. These regions thus most likely contained action-related brain activation signatures shared between actors and observers.

It could be argued that spatial smoothing and normalization could replace functional realignment by decreasing anatomical misalignment across individuals. However, here we studied whether the activity patterns associated with specific actions in actors and observers are misaligned between individuals in a multivariate fashion, which is not possible to remediate with spatial smoothing. The employed BCCA approach separates execution- and observation-specific information from shared information whereas spatial smoothing would leave this modality-specific information in the signal. In addition, we evaluated predictive accuracy for between-subjects classification with spatially smoothed and normalized data and classifier performance remained below chance level (27% for meta-analytic ROI). Therefore, the applied hyperclassification went significantly beyond spatial smoothing and, in addition to misaligned anatomical features, also allowed investigations of misaligned functional activity patterns.

Validation analysis using temporally misaligned actor’s and observer’s data or surrogate data confirmed that when realigned data lack temporal coherence, hyperclassification accuracy is below chance level, showing that functional realignment is robust against noise and requires shared information to be present between the datasets. Finally, validation using unrelated ROIs confirmed that when a region was not directly involved in action execution or observation, hyperclassification accuracy rose only barely above chance level and remained significantly below any other ROI. Together, our validation analyses show that functional realignment only works when similar stimulus- or task-dependent haemodynamic patterns exist in the realigned brains.

### Action-specific brain activation signatures for action execution and observation

Previous pattern classification studies have found action-specific neural codes for both action execution and observation in the parietal and frontal cortices of single individuals [6]. Shared action-specific activity patterns have been reported also for executed and heard actions [13] as well as for executed and observed hand actions [14]. However, the results of these studies didn’t show successful between-subjects classification of perceived and executed actions to support the hypothesis that action execution and observation rely on shared brain activation signatures across individuals. Our results from hyperclassification following functional realignment extend the previous findings by showing shared action-related information between actors and observers. This information allowed successful differentiation between observed actions using neural activation patterns within a distributed network of bilateral LOC, SMG, SPL, and precentral and postcentral cortices.

Within-subject classification from observation localizer ROI showed that action observation triggered action-specific brain activation signatures in LOC, EBA, SPL, SMG and premotor cortex in both individuals executing and observing the actions. However, between-subjects classification accuracy only barely exceeded chance level in any ROI, suggesting that the brain activation signatures for action observation and execution require more than anatomical (here functional) realignment to be consistent with each other.

### Significance of shared codes for action execution and perception

When an individual views an action, action-specific information may be translated into the viewer’s action knowledge by mapping the seen actions into one’s own motor system [3]. A system with mirroring properties provides one candidate mechanism for connecting perception and action to support inference of other’s motor goals and intentions by providing the observers with a sensorimotor framework that is shared with the actor, as proposed by the direct-matching hypothesis [26, 43]. It however is still debated whether such shared codes really reflect action simulation allowing action prediction and understanding [43, 49–52]; it is also possible that shared action-related codes would be recruited as a consequence of action understanding without motor stimulation, yet this needs to be directly addressed in future experiments.

Our results, both with localizer and meta-analytic ROIs (Tables 1 and 2) suggest that a distributed set of brain areas extending beyond the ‘core’ mirroring regions encode action-related neural activity. These results agree with earlier suggestions that mirroring systems comprise a core network and extended areas that are involved only during specific tasks (see, for example [48]). Specifically, brain circuits encoding shared representations of action execution and observation in the present study included both regions with established mirroring properties (premotor cortex, IFG, and IPL) as well as regions where no activation during own actions was reported (pSTS and LOC). The observed LOC involvement in shared encoding of action execution and observation might be due to both actors and observers having visual information about performed actions. For example, it was shown that dorsal parts of LOC (EBA) and pSTS respond to goal-directed actions and limb movements [53, 54], and neural activity in primary visual cortex is coherent with the kinematics of observed actions [55].

Importantly, the results of our control experiment with the closed-eyes actor suggest that the shared visual information does not significantly contribute to successful hyperclassification. We have also controlled for low-level visual information by removing V1 from all ROIs, some information in higher-level associative areas could still have confounded our results. The EBA/LOC ROI, used as a control area, showed accuracy only marginally above chance level, and this result was further validated in the experiment with the closed-eyes actor. Action–observation matching via functional realignment thus does not seem to be based on mere shared visual information between actors and observers.

While the spatial position where the action is performed is not completely independent of the action type, we showed that even actions executed at the same position (slap, point) were successfully discriminated from the other actions, as comparable above-chance accuracies were also achieved with inherently multiclass models: a linear model implemented in MVPA toolbox and with multinomial regression with elastic-net regularization in glmnet package (data not shown). Furthermore, successful classification of executed actions, as well as hyperclassification between seen and executed actions was achieved in the control experiment with the closed-eyes actor. Thus, hyperclassification was not driven by the seen target position only.

Finally, because the premotor cortex supports action-specific information [3, 13], we also ran a functional realignment analysis using this region only. While the classification accuracy in the premotor cortex ROI was marginally above the chance level, it increased by 17 percentage points when a more extensive set of brain regions (i.e. localizer or meta-analytic ROI) was used. The higher classification accuracy in the distributed ROI suggests that shared brain activation signatures of action execution and observation involve an extensive array of regions beyond premotor cortices.

## Conclusions

Observation and execution of action are tightly linked in the brain and share action-related neural codes. We found that haemodynamic brain activity patterns in motor and sensory regions in individuals executing and observing actions contain information that allows predicting action categories. These activity patterns contained similar action-related information, but they were not aligned when different individuals executed and observed actions. Consequently, functional realignment was required to reveal the shared neural codes that may provide basis for inference of another person’s motor goals and intentions. Distributed brain activation signatures in LOC, SPL, SMA, and precentral and postcentral cortices, including SI, SII and pSTS during action execution thus seem to contain information that, after realignment, is sufficient for predicting activity elicited by observation of a corresponding action executed by another individual.
